# Dataset on the expression level of the genes involved in the synthesis of structural molecules in carbon-deficient microalgae

**DOI:** 10.1016/j.dib.2018.09.045

**Published:** 2018-09-20

**Authors:** Cheng Qilu, Xu Ligen, Cheng Fangmin, Pan Gang, Zhou Qifa

**Affiliations:** a College of Life Sciences, Zhejiang University, Hangzhou 310058, China; b College of Agriculture and Biotechnology, Zhejiang University, Hangzhou 310058, China

## Abstract

The data presented in this article are related to the research article entitled “Bicarbonate-rich wastewater as a carbon fertilizer for culture of Dictyosphaerium sp. of a giant pyrenoid” (Cheng et al., 2018) [1]. This article provides data about the expression levels of the genes involved in the synthesis of structural molecules in the carbon-deficient algal cell and the carbon-treated algal cell, which can be helpful for analyzing the observed disruption of the structural integrity in the carbon-deficient microalgae at molecular level.

**Specifications table**

**Table:** 

Subject area	*Environmental science and Biology*
More specific subject area	*Wastewater; Algal culture and Algal physiology*
Type of data	*Table, image and figure*
How data was acquired	*Microscope and RNA-sequence*
Data format	*Raw and filtered.*
Experimental factors	*The microalgae was isolated from wastewater.*
Experimental features	*Algae culture in an incubator, microscopic observation and RNA-sequence.*
Data source location	*Hangzhou City, Zhejiang Province, China*
Data accessibility	*The data is available within this article*
Related research article	*Cheng et al. 2018. Bicarbonate-rich wastewater as a carbon fertilizer for culture of Dictyosphaerium sp. of a giant pyrenoid. Journal of Cleaner Production (in press).*

**Value of the data**

• The data provided comparative transcriptomic analysis for the related Dictyosphaerium sp. genes encoding synthesis of structural molecules in the algae cultured in the modified Hoagland+Wastewater medium and the modified Hoagland medium.
• The obtained data from the RNA-seq analysis can be used for analysis of the disruption of structural integrity in the carbon-deficient microalgae at molecular level.

## 1 Data

**Fig. 1:**
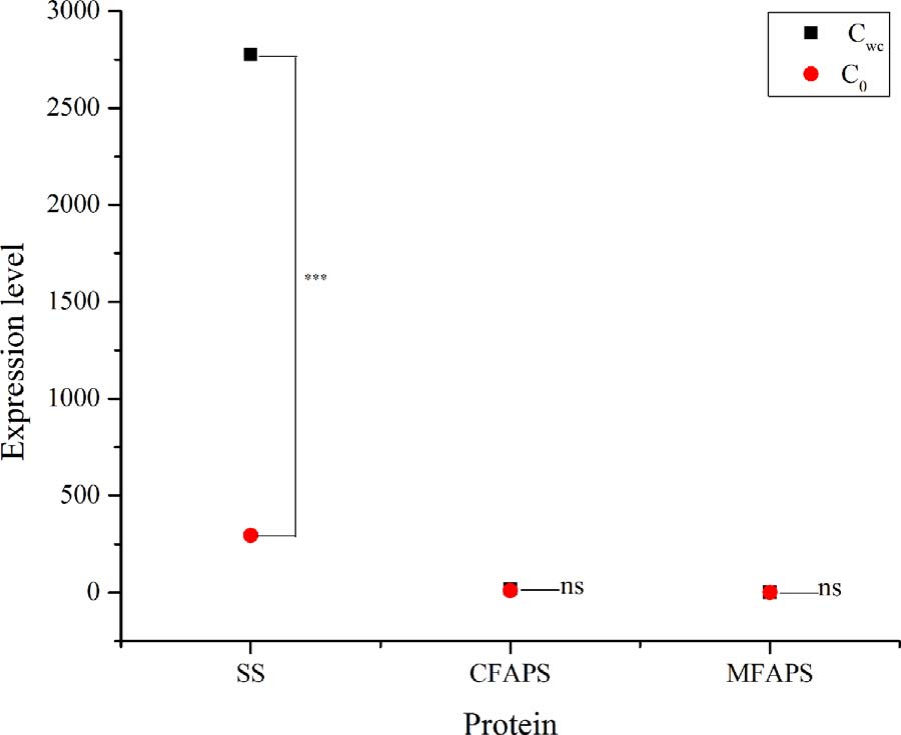
The expression level of the genes encoding the lipid synthases in the algae cultured in the modified Hoagland+Wastewater medium (C_wc_) and the Hoagland medium (C_0_). Data are means of three replications. SS- sulfolipid synthase, CFAPS- cyclopropane-fatty-acyl-phospholipid synthase, MFAPS- /methylene-fatty-acyl-phospholipid synthase.

**Fig. 2:**
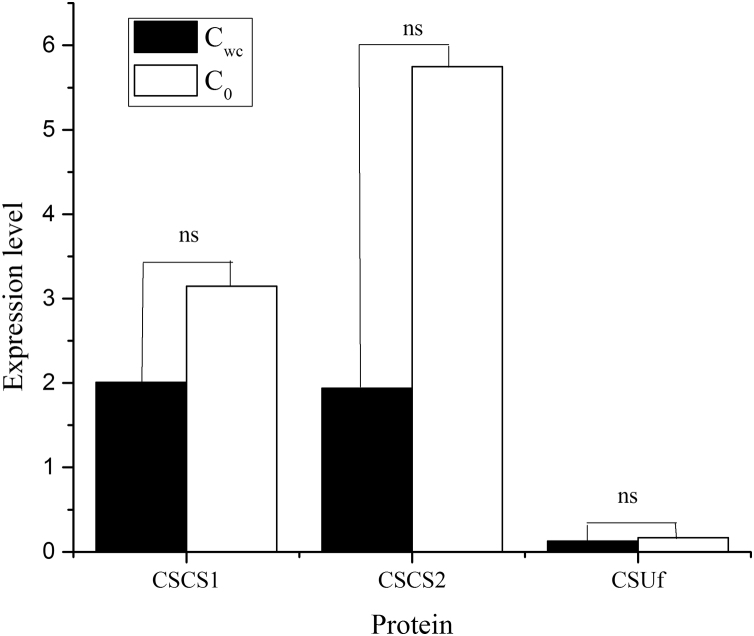
The expression level of the genes encoding cellulose synthase catalytic subunit (CSCS) and cellulose synthase (UDP-forming) (CSUf) in algae cultured in the modified Hoagland+Wastewater medium (Cwc) and the Hoagland medium (C0). Data are means of three replications. ns-non-significant (*P* > 0.05).

The dataset of this article provides information on the expression levels of the genes involved in synthesis of structural molecules in the microalgae. Table 1, Table 2, Table 3 show the expression levels of extensin protein genes, proline-rich structural protein genes, gline-rich structural protein genes, and soluble starch synthase genes and granule-bound starch synthase genes, respectively. Fig. 1, Fig. 2 show the expression levels of lipid synthase genes and cellulose synthase catalytic subunit genes and cellulose synthase genes, respectively.

**Table 1:** Comparative transcriptomic analysis for the related Dictyosphaerium sp. genes encoding extensin proteins (E-Ps) in the algae cultured in the modified Hoagland+Wastewater medium (C_wc_, Control treatment) and the modified Hoagland medium (C_0_). Data are means of three replications. L-R- leucine-rich, E-P-extensin, GI-gene ID, length-gene length, log_2_FC-log2 transformed fold change between control and treat samples, Padj- Statistic of adjusted pvalue (DEseq. 2 method used).

GI	Length(bp)	Protein	C_wc_-EL	C_0-_EL	log_2_FC	Padj
XP_011043443.1	502	L-R repeat E-P 4	0.43	1.50	1.81	0.44
XP_014660371.1	867	E-P-like	8.81	0.26	−5.07	0.016
NP_001147655.1	303	E-P	0.26	1.15	2.13	0.40
XP_010691715.1	1029	L-R repeat E-P 3	210.29	1447.15	2.78	2.76E−10
XP_008224469.1	733	L-R repeat E-P 3	0.16	0.11	−0.62	0.76
XP_009113077.1	2139	L-R repeat E-P 2 isoform X1	1239.01	234.56	−2.40	5.99E−38
XP_010691715.1	485	L-R repeat E-P 3	241.40	385.51	0.68	0.12
XP_014660371.1	841	E-P-like	0.47	39.44	6.40	5.98E−07
XP_010691715.1	1067	L-R repeat E-P 3	240.82	6.79	−5.15	0.025
XP_014660371.1	2004	E-P-like	226.18	11.70	−4.27	1.41E−06
XP_014660371.1	1889	E-P-like	203.96	364.59	0.84	0.23
XP_010257306.1	475	pollen-specific L-R repeat E-P3 isoform X1	0.15	3.59	4.62	0.032
AAX82555.1	328	120 kDa pistil E-P	0.15	1.28	3.07	0.21
XP_014660371.1	3448	E-P-like	3.86	101.83	4.72	9.29E−05
XP_010691715.1	1096	L-R repeat E-P 3	545.66	660.30	0.28	0.76
XP_011463721.1	310	E-P−2-like, partial	2.34	0.19	−3.65	0.12
XP_014660371.1	633	E-P-like	1.64	140.50	6.42	7.91E−14
XP_014660371.1	4382	E-P-like	0.18	2.62	3.87	0.096
XP_015386198.1	1600	pollen-specific L-R repeat E-P 3	23.92	291.52	3.61	2.22E−15
XP_015078119.1	718	E-P−1-like	46.35	229.17	2.31	1.14E−11
XP_008224469.1	797	L-R repeat E-P 3	0.18	21.89	6.94	9.80E−05
XP_004287084.1	894	L-R repeat E-P 4	20.18	221.26	3.45	2.30E−05
XP_009415697.1	310	L-R repeat E-P 4	0.37	2.12	2.53	0.19
XP_013599358.1	1539	L-R repeat E-P 5 isoform X3	448.84	166.85	−1.43	1.27E−08
XP_012464092.1	765	L-R repeat E-P 1	6.18	149.41	4.59	5.35E−11
XP_014660371.1	1250	E-P like	0.42	11.64	4.79	0.00087
XP_014660371.1	3854	E-P-like	3.17	4.36	0.46	0.83
XP_008224469.1	591	L-R repeat E-P 3	0.38	0.97	1.35	0.58
XP_014660371.1	1810	E-P-like	9.72	206.32	4.41	1.80E−16
XP_014660371.1	1271	E-P-like	15.53	856.18	5.78	3.47E−08
XP_008675925.1	1338	E-P-like	3.86	40.68	3.40	4.48E−08
XP_014660371.1	1447	E-P-like	0.21	25.68	6.94	0.00014
CAA46283.1	1156	E-P	0.24	0.87	1.86	0.46
XP_009348443.1	446	L-R repeat E-P 4	0.16	7.60	5.57	0.0051
XP_011093910.1	766	L-R repeat E-P 1	1.74	22.06	3.67	1.57E−05
XP_014660371.1	1383	E-P-like	3.96	125.73	4.99	2.01E−13
CAA46283.1	936	E-P	335.08	6725.57	4.33	1.44E−16
XP_010691715.1	991	L-R repeat E-P 3	630.93	2372.68	1.91	0.0022
XP_006846406.2	221	PR-P E-P EPR1	0.21	0.13	−0.75	0.72
XP_008803695.1	438	L-R repeat E-P 3	110.48	10.71	−3.37	7.27E−15
XP_015057744.1	1063	E-P-like	2.77	39.43	3.83	1.58E−07
XP_010449482.1	248	L-R repeat E-P 5	0.29	0.33	0.18	0.94
XP_008224469.1	986	L-R repeat E-P 3	1.80	100.62	5.80	3.67E−18
XP_008224469.1	425	L-R repeat E-P 3	0.23	0.83	1.83	0.47
XP_014660371.1	1163	E-P-like	26.67	224.01	3.07	0.00062
XP_014660371.1	1442	E-P-like	115.01	9.44	−3.61	0.14
XP_014660371.1	3696	E-P-like	0.26	9.77	5.24	0.014
XP_014660371.1	2040	E-P-like	2.65	183.45	6.12	3.25E−10
XP_004242070.1	267	E-P-like	4.43	8.31	0.91	0.34
XP_010249819.1	1621	L-R repeat E-P 4	1.47	0.15	−3.31	0.16
XP_010262231.1	228	L-R repeat E-P 3	0.15	1.28	3.07	0.21
XP_002519151.1	2314	pollen-specific L-R repeat E-P 1	1311.14	635.98	−1.04	0.00019
XP_012444558.1	428	pistil-specific E-P	0.16	0.11	−0.62	0.76
XP_014660371.1	3994	E-P-like	0.23	6.46	4.80	0.028
AFW77096.1	482	PR-P E-P-like receptor protein kinase family protein	0.12	1.43	3.56	0.13
XP_014509417.1	218	L-R repeat E-P 4	0.86	0.26	−1.72	0.50
XP_009376329.1	727	L-R repeat E-P 4	109.69	350.83	1.68	0.0046
XP_010448887.1	7218	PR-P E-P EPR1	56,642.06	76,119.34	0.43	0.17
XP_009111855.1	319	E-P-like	0.13	2.11	3.97	0.081
XP_015057744.1	831	E-P-like	0.15	5.85	5.26	0.0099
XP_014660371.1	1605	E-P-like	1.30	0.31	−2.05	0.42
XP_014660371.1	1672	E-P-like	3.69	86.21	4.55	1.19E−14
XP_010449482.1	477	L-R repeat E-P 5	0.40	5.55	3.81	0.020
XP_013743627.1	950	pollen-specific L-R repeat E-P 1	148.85	286.64	0.95	0.18
XP_008224469.1	1413	L-R repeat E-P 3	306.51	31.40	−3.29	8.98E−26
XP_007013165.1	346	PR-P E-P-like receptor kinase 1	0.71	35.61	5.65	2.91E−07
XP_014660371.1	3836	E-P-like	0.17	13.30	6.30	0.00075
XP_014660371.1	887	E-P-like	0.71	33.83	5.58	5.59E−07
XP_006480071.1	249	pollen-specific L-R repeat E-P 4	0.48	0.20	−1.25	0.61
XP_008224469.1	2210	L-R repeat E-P 3	3.76	626.49	7.38	2.57E−16
XP_014660371.1	779	E-P-like	0.68	2.66	1.97	0.36
XP_010647090.1	377	E-P-like	0.19	16.62	6.42	0.00067
XP_009348443.1	1192	L-R repeat E-P 4	178.48	127.98	−0.48	0.081
XP_014660371.1	711	E-P-like	42.98	3.81	−3.50	0.15
CAA84230.1	526	E-P-like protein	0.13	2.14	4.04	0.072
XP_008224469.1	246	L-R repeat E-P 3	0.29	0.15	−0.94	0.69
XP_009348443.1	972	L-R repeat E-P 4	111.37	30.23	−1.88	2.20E−07
XP_008670898.1	850	pollen-specific L-R repeat E-P 3	0.18	15.61	6.42	0.00061
CAA46283.1	858	E-P	0.22	4.60	4.42	0.050
XP_014660371.1	923	E-P-like	3.63	0.64	−2.50	0.24
XP_015057744.1	562	E-P-like	0.87	7.47	3.11	0.13
XP_014660371.1	690	E-P-like	0.67	7.22	3.43	0.014
XP_009114442.1	457	L-R repeat E-P 3	45.54	16.77	−1.44	0.046
XP_015388802.1	461	PR-P E-P EPR1	1.41	6.04	2.09	0.074
CAA46283.1	899	E-P	1.23	28.37	4.53	1.84E−06
XP_008378297.1	1476	L-R repeat E-P 3	920.74	421.90	−1.13	0.00025
XP_008782559.1	243	Low quality protein:E-P−2-like	0.12	0.60	2.33	0.36
XP_010088853.1	928	L-R repeat E-P 4	2.10	33.48	4.00	1.17E−07
XP_013660711.1	408	L-R repeat E-P 2	17.69	9.91	−0.84	0.24
XP_014660371.1	3635	E-P-like	7.04	0.57	−3.63	0.13
CAA46283.1	872	E-P	0.15	4.90	5.04	0.015
NP_001147655.1	344	E-P	0.18	2.12	3.55	0.14
XP_012078651.1	322	L-R repeat E-P 4	0.13	0.09	−0.52	0.77
AFW77096.1	1224	PR-P E-P-like receptor protein kinase family protein	0.17	11.28	6.07	0.0015
XP_008224469.1	553	L-R repeat E-P 3	176.48	44.53	−1.99	2.49E−09
CAA46283.1	789	E-P	1.53	35.14	4.52	3.57E−08
XP_010270865.1	1253	L-R repeat E-P 3 isoform X2	2.01	62.31	4.95	1.03E−09
XP_009135898.1	643	L-R repeat E-P 4	0.14	4.02	4.84	0.021
XP_008645230.1	861	L-R repeat E-P 7	2.38	7.12	1.58	0.27
XP_014660371.1	395	E-P-like	3.84	0.20	−4.25	0.054
XP_008441528.1	513	E-P−1-like isoform X1	18.53	1.39	−3.74	0.00096
XP_008224469.1	1260	L-R repeat E-P 3	1.20	27.88	4.53	1.27E−06
XP_006494306.1	957	pollen-specific L-R repeat E-P 3	66.57	47.63	−0.48	0.22
XP_008224469.1	663	L-R repeat E-P 3	5.15	15.52	1.59	0.16
XP_008677910.1	437	pollen-specific L-R repeat E-P 3	1.79	5.08	1.51	0.27
XP_014660371.1	837	E-P-like	0.63	10.10	4.01	0.034
XP_014660371.1	572	E-P-like	33.48	0.66	−5.67	0.0081
XP_014660371.1	1759	E-P-like	31.36	0.45	−6.14	0.0024
XP_014660371.1	3308	E-P-like	8.43	35.22	2.06	0.077
XP_010439825.1	1046	L-R repeat E-P 4	0.15	0.31	1.03	0.66
XP_009135898.1	1270	L-R repeat E-P 4	414.40	49.48	−3.07	2.34E−17
XP_010249819.1	799	L-R repeat E-P 4	0.90	6.72	2.90	0.026
XP_010691715.1	1134	L-R repeat E-P 3	335.55	0.33	−10.00	1.52E−11
XP_002518703.2	822	L-R repeat E-P 3	422.05	314.10	−0.43	0.31
XP_009402812.1	279	L-R repeat E-P 3	0.12	0.60	2.33	0.36

**Table 2:** Comparative transcriptomic analysis for the related *Dictyosphaerium sp.* genes encoding gline-rich structural proteins (GR-Ps) in the algae cultured in the modified Hoagland+Wastewater medium (C_wc_, Control treatment) and the Hoagland medium (C_0_). Data are means of three replications. GI-gene ID, length-gene length, log_2_FC-log2 transformed fold change between control and treat samples, Padj- Statistic of adjusted p value (DEseq. 2 method used).

GI	Length(bp)	Protein	Cwc-EL	C0-EL	log_2_FC	Padj
XP_015574762.1	351	GR-P-like	0.13	0.17	0.42	0.83
XP_006651317.1	1054	GR-P-like	1.41	2.92	1.05	0.70
XP_008666361.1	444	GR-P 1.0-like	0.25	0.27	0.11	0.97
XP_010466892.1	514	GR-P 1-like	0.23	0.39	0.77	0.77
XP_010466892.1	497	GR-P 1-like	0.60	0.81	0.42	0.87
XP_013681405.1	480	GR-P 1.8-like isoform X1	24.21	138.83	2.52	0.33
XP_015574762.1	841	GR-P-like	0.35	3.03	3.10	0.20
XP_012570960.1	630	GR-P-like isoform X6	0.24	2.46	3.34	0.15
XP_015574762.1	4268	GR-P-like	0.23	0.39	0.77	0.77
XP_015166590.1	510	GR-P 1.8-like	0.13	0.17	0.42	0.83
XP_010467102.1	722	GR-P 1.0-like isoform X10	5.08	11.83	1.22	0.29
XP_015167339.1	3234	GR-P 1	73.83	153.22	1.05	0.43
XP_010688958.1	854	GR-P-like isoform X1	8.55	52.39	2.62	0.012
XP_009143614.1	249	GR-P 1.8	1.09	1.83	0.75	0.80
XP_009143614.1	594	GR-P 1.8	1.69	7.46	2.14	0.13
XP_013668804.1	1666	GR-P 1-like	0.97	1.97	1.02	0.66
XP_013731827.1	1381	GR-P-like	349.42	289.25	−0.27	0.77
XP_010424670.1	359	GR-like	0.22	0.37	0.76	0.77
XP_008671409.1	1553	GR-P 1.0-like	16.78	103.53	2.63	0.00082
XP_008671490.1	385	GR-P 1.8-like	0.18	1.01	2.45	0.35
XP_010467104.1	890	GR-P 1.0-like isoform X12	0.50	2.75	2.44	0.29
XP_010466892.1	853	GR-P 1-like	125.17	288.18	1.20	0.15
XP_014628139.1	392	GR-P 1.8	27.57	16.66	−0.73	0.58
XP_010467093.1	1047	GR-P 1.8-like isoform X1	99.08	5.45	−4.19	0.0052
XP_014628139.1	369	GR-P 1.8	57.90	52.71	−0.14	0.88
XP_015580618.1	923	GR-P 1.8 isoform X2	0.21	0.34	0.71	0.78
XP_011465792.1	254	GR-P 1.8	38.54	28.54	−0.43	0.77
XP_012570962.1	906	GR-P-like isoform X10	61.95	77.82	0.33	0.77
XP_010317299.1	478	putative GR-P 1	0.37	0.88	1.25	0.66
XP_015389986.1	418	putative GR-P 1	0.16	0.41	1.39	0.63
XP_006490141.1	952	GR-P-like	1173.51	5692.52	2.28	6.05E−07
XP_008229859.1	950	GR-P	1.82	2.83	0.64	0.77
XP_010466892.1	554	GR-P 1-like	45.86	40.86	−0.17	0.85
XP_010432984.1	328	putative GR-P 1	0.21	0.68	1.71	0.54
XP_010467093.1	1149	GR-P 1.8-like isoform X1	29.54	0.36	−6.35	0.00014
XP_010456984.1	1304	GR-P 1-like	0.83	0.23	−1.84	0.51
XP_012570964.1	1671	GR-P-like isoform X12	0.29	0.29	−0.02	0.99
XP_009336768.1	1071	putative GR-P 1	186.65	605.46	1.70	0.092
XP_015574762.1	707	GR-P-like	0.16	0.41	1.39	0.63
XP_010424670.1	454	GR-P-like	0.66	0.62	−0.10	0.98
XP_010680074.1	1244	GR-P-like	2.88	22.39	2.96	0.014
P10496.1	998	GR-P 1.8; Short=GRP 1.8;Flags: Precursor	282.59	1011.12	1.84	0.00066
XP_010424670.1	925	GR-P-like	0.73	3.77	2.36	0.25
XP_012570964.1	473	GR-P-like isoform X12	76.32	100.18	0.39	0.65

**Table 3:** Comparative transcriptomic analysis for the related *Dictyosphaerium sp.* genes encoding soluble starch synthase (SSS) and granule-bound starch synthase (GBSS) in the algae cultured in the modified Hoagland+Wastewater medium (C_wc_, Control treatment) and the modified Hoagland medium (C0). Data are means of three replications. GI-gene ID, length-gene length, log2FC-log2 transformed fold change between control and treat samples, Padj- Statistic of adjusted pvalue (DEseq. 2 method used).

GI	Length(bp)	Protein	Cwc-EL	C0-EL	log_2_FC	Padj
BAE79814.1	376	GBSS	1043.71	236.79	−2.14	4.30E−12
XP_011398759.1	5924	SSS 3, chloroplastic/amyloplastic	2879.90	930.15	−1.63	2.71E−15
BAE79814.1	537	GBSS	3.01	223.15	6.21	3.60E−17
XP_001697117.1	1674	GBSS-I	559.29	1833.07	1.71	0.014
BAE79814.1	562	GBSS	0.95	12.94	3.76	0.0015
BAE79814.1	292	GBSS	744.96	148.47	−2.33	4.62E−17
BAE79814.1	292	GBSS	744.96	148.47	−2.33	4.62E−17
BAE79814.1	2113	GBSS	4963.46	2460.34	−1.01	4.80E−05
XP_011401562.1	2260	putative GBSS 1, chloroplastic/amyloplastic	2.83	82.37	4.86	4.57E−13
BAE79814.1	562	GBSS	0.95	12.94	3.76	0.0015
XP_001697117.1	1030	GBSS-I	49.21	94.50	0.94	0.041
BAE79814.1	376	GBSS	1043.71	236.79	−2.14	4.30E−12
BAE79814.1	4024	GBSS	1101.80	2197.76	1.00	0.011
BAE79814.1	537	GBSS	3.01	223.15	6.21	3.60E−17
XP_001697117.1	1674	GBSS-I	559.29	1833.07	1.71	0.014
BAE79814.1	2113	GBSS	4963.46	2460.34	−1.01	4.80E−05
XP_011398759.1	6692	SSS 3, chloroplastic/amyloplastic	12.72	384.67	4.92	2.36E−22
XP_005642568.1	375	SSS	14.16	1.74	−3.02	0.0026
XP_011398759.1	496	SSS 3, chloroplastic/amyloplastic	41.16	9.23	−2.16	0.00011
AAC17970.2	259	SSS	9.10	8.88	−0.036	0.97
XP_011398759.1	6692	SSS 3, chloroplastic/amyloplastic	12.72	384.67	4.92	2.36E−22
XP_005642568.1	375	SSS	14.16	1.74	−3.02	0.0026
XP_001695327.1	248	SSS- III	4.19	4.55	0.12	0.92
XP_011398759.1	5924	SSS 3, chloroplastic/amyloplastic	2879.90	930.15	−1.63	2.71E−15
XP_011398759.1	496	SSS 3, chloroplastic/amyloplastic	41.16	9.236	−2.16	0.00011

## 2 Experimental design, materials, and methods

The green algae strain Dictyosphaerium sp. was isolated from wastewater originating from the Experimental Farm at Zhejiang University. Prior to cultivation experiments, the species was cultured in BG11 medium until reaching log phase. A batch of algal culture experiments were conducted in a model ACM−168 algal incubator (Jiangnan Instrument Co., Ningbo, China). Two treatments with three replications were conducted with 0 and 0.25 L/L of the autoclaved swine wastewater to modified Hoagland solution [2], and the wastewater-added medium contained 0.8 g/L bicarbonate. Microalgae seeds were added to different culture media in 250 mL triangular glass flasks with 200 mL of working solution, and with initial optical density values adjusted to between 0.05 and 0.07 absorbance at a wavelength of 680 nm (OD_680_).

RNA sampling and extraction was performed according to methods described in [3]. On the 13th day, 10 mL of cultures were collected by centrifugation (10,000 g, 7 min) from each replicate. The supernatant was discarded and the resulting cell pellets were immediately flash-frozen with liquid nitrogen and stored at −80 °C. Prior to RNA extraction, cell pellets were resuspended in lysis buffer and then ground using a micropestle. Total RNA was then extracted following the manufacturer׳s instructions. Total RNA was maintained as replicates, rather than pooling, and then stored at −80 °C. An Agilent 2100 Bio analyzer (Agilent RNA 6000 Nano Kit) was used to determine QC:RNA concentrations, RIN values, 28 S/18 S, and fragment length distributions. A NanoDropTM spectrophotometer was used to assess the purity of the RNA. Aliquots from mRNA samples were used to construct cDNA libraries.

After quality filtering sequence reads, clean reads were mapped to the genomic reference using Bowtie2 [2], and gene expression levels were calculated with RSEM [4]. Differentially expressed genes (DEGs) were determined with DEseq. 2 [5].

## References

[1] Cheng Q.L., Xu L.G., Cheng F.M., pan G., Zhou Q.F. (2018). Bicarbonate-rich wastewater as a carbon fertilizer for culture of Dictyosphaerium sp. of a giant pyrenoid. J. Clean. Prod..

[2] Zhang J., Wang X., Zhou Q. (2017). Co-cultivation of Chlorella spp and tomato in a hydroponic system. Biomass-. Bioenergy.

[3] Langmead B., Salzberg S.L. (2012). Fast gapped-read alignment with Bowtie 2. Nat. Methods.

[4] Li B., Dewey C.N. (2011). RSEM: accurate transcript quantification from RNA-Seq data with or without a reference genome. BMC Bioinform..

[5] Love M.I., Huber W., Anders S. (2014). Moderated estimation of fold change and dispersion for RNA-seq data with DESeq. 2. Genome Biol..

