# Supplementary material for: Dataset on the expression level of the genes involved in the synthesis of structural molecules in carbon-deficient microalgae
Source: Data Brief. 2018 Sep 20;20:1870–6. doi: 10.1016/j.dib.2018.09.045 (PMC6168790; doi:10.1016/j.dib.2018.09.045)
Supplement: Supplementary file 1 — Supplementary material [file mmc1.docx]

Conflict of interests statement

The authors declare no conflict of interests.
