# Supplementary material for: Dataset on the expression level of the genes involved in the synthesis of structural molecules in carbon-deficient microalgae
Source: Data Brief. 2018 Sep 20;20:1870–6. doi: 10.1016/j.dib.2018.09.045 (PMC6168790; doi:10.1016/j.dib.2018.09.045)
Supplement: Supplementary file 2 — Supplementary material [file mmc2.docx]

**Table 2**

Comparative transcriptomic analysis for the related *Dictyosphaerium sp.* genes encoding proline-rich structural protein (PR-Ps) in the algae cultured in the modified Hoagland+Wastewater medium (Cwc, Control treatment) and the Hoagland medium (C0). Data are means of three replications. GI-gene ID, length-gene length, log2FC-log2 transformed fold change between control and treat samples, Padj- Statistic of adjusted pvalue (DEseq2 method used).

| GI | Length(bp) | Protein | Cwc-EL | C0-EL | log_2_FC | Padj |
| --- | --- | --- | --- | --- | --- | --- |
| XP_015067866.1 | 3294 | basic PR-P-like isoform X1 | 10.25 | 307.88 | 4.91 | 4.10E-09 |
| XP_010644381.1 | 548 | basic PR-P-like | 0.64 | 3.77 | 2.57 | 0.095 |
| XP_015067866.1 | 3977 | basic PR-P-like isoform X1 | 1507.74 | 1196.64 | -0.33 | 0.086 |
| XP_015067866.1 | 527 | basic PR-P-like isoform X1 | 0.17 | 16.23 | 6.56 | 0.00034 |
| XP_015067866.1 | 2367 | basic PR-P-like isoform X1 | 11.56 | 357.59 | 4.95 | 0.0071 |
| XP_015067866.1 | 2149 | basic PR-P-like isoform X1 | 233.88 | 164.33 | -0.51 | 0.041 |
| XP_008660804.1 | 1611 | basic PR-P-like | 363.28 | 240.37 | -0.60 | 0.055 |
| XP_015067866.1 | 721 | basic PR-P-like isoform X1 | 0.40 | 5.14 | 3.69 | 0.029 |
| XP_014755416.1 | 888 | basic PR-P-like | 615.96 | 659.76 | 0.10 | 0.77 |
| XP_006362904.1 | 321 | basic PR-P | 0.14 | 0.94 | 2.77 | 0.26 |
| XP_010477244.1 | 1030 | anther-specific PR-P APG | 1.10 | 3.59 | 1.71 | 0.34 |
| XP_006339345.1 | 1350 | anther-specific PR-P APG-like | 215.88 | 385.77 | 0.84 | 0.15 |
| XP_015067866.1 | 408 | basic PR-P-like isoform X1 | 104.98 | 25.19 | -2.06 | 1.16E-06 |
| XP_010477244.1 | 231 | anther-specific PR-P APG | 0.40 | 0.88 | 1.13 | 0.68 |
| XP_015067866.1 | 785 | basic PR-P-like isoform X1 | 0.17 | 15.52 | 6.49 | 0.00044 |
| XP_008680071.1 | 361 | anther-specific PR-P APG-like | 15.84 | 11.84 | -0.42 | 0.63 |
| XP_015067867.1 | 2182 | basic PR-P-like isoform X2 | 15.48 | 232.52 | 3.91 | 1.14E-20 |
| XP_008660804.1 | 1369 | basic PR-P-like | 0.17 | 17.70 | 6.74 | 0.00017 |
| XP_015067866.1 | 1546 | basic PR-P-like isoform X1 | 4.87 | 97.26 | 4.32 | 1.39E-06 |
| XP_014755416.1 | 261 | basic PR-P-like | 0.14 | 4.74 | 5.03 | 0.015 |
| XP_008653760.1 | 1699 | basic PR-P-like | 564.82 | 1870.69 | 1.73 | 3.26E-11 |
| XP_015067866.1 | 2865 | basic PR-P-like isoform X1 | 1092.39 | 225.54 | -2.28 | 3.72E-12 |
| XP_015067866.1 | 1212 | basic PR-P-like isoform X1 | 0.97 | 25.64 | 4.72 | 2.81E-06 |
| XP_015067866.1 | 1503 | basic PR-P-like isoform X1 | 370.25 | 195.67 | -0.92 | 0.0011 |
| XP_015067866.1 | 597 | basic PR-P-like isoform X1 | 0.15 | 8.28 | 5.77 | 0.0029 |
| XP_015067867.1 | 3756 | basic PR-P-like isoform X2 | 4.63 | 69.92 | 3.92 | 9.24E-06 |
| XP_015067866.1 | 1861 | basic PR-P-like isoform X1 | 1.33 | 9.49 | 2.84 | 0.16 |
| XP_015067866.1 | 1782 | basic PR-P-like isoform X1 | 3.29 | 39.53 | 3.59 | 3.29E-08 |
| XP_015067866.1 | 1031 | basic PR-P-like isoform X1 | 1.97 | 21.71 | 3.46 | 6.26E-05 |
| XP_014755416.1 | 1184 | basic PR-P-like | 3.14 | 13.57 | 2.11 | 0.035 |
| XP_015067866.1 | 2495 | basic PR-P-like isoform X1 | 46.19 | 1756.95 | 5.25 | 3.72E-31 |
| XP_015067867.1 | 567 | basic PR-P-like isoform X2 | 0.46 | 36.10 | 6.30 | 1.36E-06 |
| XP_015067866.1 | 1150 | basic PR-P-like isoform X1 | 0.50 | 4.11 | 3.05 | 0.14 |
| XP_008660804.1 | 1864 | basic PR-P-like | 5.52 | 506.99 | 6.52 | 1.74E-36 |
| XP_015067866.1 | 1673 | basic PR-P-like isoform X1 | 0.13 | 0.70 | 2.43 | 0.34 |
| XP_008660804.1 | 3169 | basic PR-P-like | 19.14 | 664.48 | 5.12 | 2.23E-45 |
| XP_015067866.1 | 2591 | basic PR-P-like isoform X1 | 499.61 | 163.68 | -1.61 | 3.44E-07 |
| XP_015067866.1 | 4451 | basic PR-P-like isoform X1 | 95.09 | 4376.27 | 5.52 | 5.69E-22 |
| XP_010313594.1 | 319 | basic PR-P, partial | 0.18 | 3.42 | 4.21 | 0.063 |
| XP_015067866.1 | 335 | basic PR-P-like isoform X1 | 0.15 | 6.18 | 5.34 | 0.0082 |
| XP_015067866.1 | 1231 | basic PR-P-like isoform X1 | 446.72 | 132.54 | -1.75 | 3.30E-09 |
| XP_008660804.1 | 1106 | basic PR-P-like | 0.63 | 1.07 | 0.76 | 0.75 |
| XP_015067866.1 | 4877 | basic PR-P-like isoform X1 | 20.99 | 226.75 | 3.43 | 1.25E-20 |
| XP_015067866.1 | 2755 | basic PR-P-like isoform X1 | 3.16 | 23.01 | 2.86 | 0.015 |
| XP_015067866.1 | 2460 | basic PR-P-like isoform X1 | 2.60 | 138.97 | 5.74 | 4.28E-11 |
| XP_015067867.1 | 1653 | basic PR-P-like isoform X2 | 5.56 | 117.38 | 4.40 | 0.00014 |
| XP_008660804.1 | 902 | basic PR-P-like | 240.63 | 130.22 | -0.89 | 0.0027 |
| XP_015067866.1 | 616 | basic PR-P-like isoform X1 | 0.44 | 25.76 | 5.86 | 9.91E-06 |
| XP_014755416.1 | 943 | basic PR-P-like | 4.96 | 196.99 | 5.31 | 4.79E-24 |
| XP_008660804.1 | 1493 | basic PR-P-like | 1.25 | 58.87 | 5.56 | 1.21E-11 |
| XP_015067866.1 | 2013 | basic PR-P-like isoform X1 | 0.68 | 8.76 | 3.69 | 0.013 |
| XP_008660804.1 | 1326 | basic PR-P-like | 2.04 | 32.01 | 3.97 | 2.31E-06 |
| XP_008660804.1 | 1183 | basic PR-P-like | 1.52 | 6.26 | 2.04 | 0.20 |
| XP_010477244.1 | 801 | anther-specific PR-P APG | 31.70 | 6.81 | -2.22 | 0.00050 |
| XP_015067866.1 | 3036 | basic PR-P-like isoform X1 | 1.51 | 68.71 | 5.51 | 5.91E-13 |
| XP_015067866.1 | 861 | basic PR-P-like isoform X1 | 0.43 | 20.76 | 5.60 | 3.72E-05 |
| XP_008660804.1 | 1765 | basic PR-P-like | 1.22 | 139.94 | 6.84 | 1.52E-08 |
| XP_010477244.1 | 366 | anther-specific PR-P APG | 0.18 | 23.11 | 6.97 | 9.51E-05 |
| XP_010477244.1 | 786 | anther-specific PR-P APG | 101.30 | 81.60 | -0.31 | 0.50 |
| XP_008660804.1 | 1479 | basic PR-P-like | 0.74 | 7.96 | 3.43 | 0.027 |
| XP_015067866.1 | 1624 | basic PR-P-like isoform X1 | 0.43 | 3.58 | 3.06 | 0.10 |
| XP_015067866.1 | 2611 | basic PR-P-like isoform X1 | 0.23 | 184.05 | 9.67 | 1.75E-10 |
| XP_015067866.1 | 2747 | basic PR-P-like isoform X1 | 4.72 | 7.30 | 0.63 | 0.77 |
| XP_015067866.1 | 1523 | basic PR-P-like isoform X1 | 0.14 | 2.70 | 4.27 | 0.053 |
| XP_006362904.1 | 1442 | basic PR-P | 139.77 | 81.18 | -0.78 | 0.041 |
| XP_014755416.1 | 409 | basic PR-P-like | 0.44 | 10.99 | 4.65 | 0.0013 |
| XP_015067866.1 | 2340 | basic PR-P-like isoform X1 | 9.99 | 673.20 | 6.08 | 1.03E-37 |
| XP_010477244.1 | 289 | anther-specific PR-P APG | 0.36 | 0.17 | -1.07 | 0.66 |
| XP_008660804.1 | 653 | basic PR-P-like | 0.41 | 9.95 | 4.59 | 0.0023 |
| XP_015067866.1 | 2816 | basic PR-P-like isoform X1 | 6.90 | 61.71 | 3.16 | 0.18 |
| XP_008660804.1 | 763 | basic PR-P-like | 0.11 | 1.07 | 3.23 | 0.18 |
| XP_014755416.1 | 409 | basic PR-P-like | 0.53 | 1.82 | 1.79 | 0.48 |
| XP_015067867.1 | 1121 | basic PR-P-like isoform X2 | 0.41 | 4.57 | 3.47 | 0.056 |
| XP_008660804.1 | 2153 | basic PR-P-like | 7.04 | 314.20 | 5.48 | 1.25E-29 |
| XP_015067866.1 | 1073 | basic PR-P-like isoform X1 | 0.13 | 1.76 | 3.78 | 0.10 |
| XP_015067866.1 | 971 | basic PR-P-like isoform X1 | 2.57 | 69.28 | 4.75 | 3.94E-13 |
| XP_008356297.1 | 933 | basic PR-P-like | 7.91 | 149.43 | 4.24 | 1.48E-24 |
| XP_010477244.1 | 301 | anther-specific PR-P APG | 0.69 | 36.42 | 5.72 | 3.89E-08 |
| XP_015067866.1 | 1306 | basic PR-P-like isoform X1 | 36.28 | 115.43 | 1.67 | 0.0024 |
| XP_015067866.1 | 1448 | basic PR-P-like isoform X1 | 740.66 | 5037.29 | 2.77 | 7.43E-05 |
| XP_014755416.1 | 573 | basic PR-P-like | 108.94 | 79.30 | -0.46 | 0.089 |
| XP_010477244.1 | 651 | anther-specific PRP APG | 0.13 | 1.87 | 3.86 | 0.091 |
| XP_008653208.1 | 1561 | PR-P HaeIII subfamily 1-like isoform X2 | 24.60 | 531.29 | 4.43 | 1.95E-40 |
| XP_015067866.1 | 2472 | basic PR-P-like isoform X1 | 0.26 | 13.34 | 5.68 | 0.0061 |
| XP_010477244.1 | 632 | anther-specific PR-P APG | 1.23 | 21.45 | 4.12 | 2.96E-05 |
| XP_015067866.1 | 2365 | basic PR-P-like isoform X1 | 13.24 | 413.59 | 4.97 | 1.45E-19 |
| XP_013584339.1 | 642 | anther-specific PR-P APG | 0.12 | 0.60 | 2.33 | 0.36 |
| XP_008660804.1 | 794 | basic PR-P-like | 0.16 | 5.15 | 5.01 | 0.017 |
| XP_008680864.1 | 927 | basic PR-P-like | 0.44 | 18.27 | 5.37 | 0.00012 |
| XP_008660804.1 | 1514 | basic PR-P-like | 0.19 | 44.87 | 7.85 | 2.98E-06 |
| XP_015067867.1 | 3820 | basic PR-P-like isoform X2 | 5.26 | 271.40 | 5.69 | 1.35E-15 |
| XP_010644788.1 | 706 | basic PR-P-like | 0.95 | 25.78 | 4.76 | 1.14E-06 |
| XP_008660804.1 | 645 | basic PR-P-like | 22.72 | 18.59 | -0.29 | 0.75 |
| XP_015067866.1 | 4217 | basic PR-P-like isoform X1 | 1649.64 | 913.28 | -0.85 | 1.07E-06 |
| XP_013640092.1 | 511 | 36.4 kDa PR-P-like isoform X1 | 1.82 | 0.21 | -3.14 | 0.20 |
| XP_015067867.1 | 2094 | basic PR-P-like isoform X2 | 4.53 | 140.18 | 4.95 | 8.09E-15 |
| XP_014755416.1 | 2334 | basic PR-P-like | 776.67 | 1624.77 | 1.06 | 2.81E-05 |
| XP_008660804.1 | 933 | basic PR-P-like | 1.78 | 33.35 | 4.23 | 2.32E-06 |
| XP_015067866.1 | 1138 | basic PR-P-like isoform X1 | 0.19 | 38.29 | 7.66 | 6.25E-06 |
| XP_015067866.1 | 1509 | basic PR-P-like isoform X1 | 11.75 | 178.41 | 3.92 | 5.87E-12 |
| XP_008660804.1 | 1796 | basic PR-P-like | 365.07 | 96.86 | -1.91 | 3.05E-13 |
| XP_015067866.1 | 2880 | basic PR-P-like isoform X1 | 2.52 | 111.01 | 5.46 | 2.07E-06 |
| XP_015067866.1 | 1877 | basic PR-P-like isoform X1 | 351.82 | 321.44 | -0.13 | 0.70 |
| XP_015067866.1 | 4114 | basic PR-P-like isoform X1 | 1092.12 | 761.15 | -0.52 | 0.0019 |
| XP_008660804.1 | 258 | basic PR-P-like | 0.42 | 9.03 | 4.43 | 0.0031 |
| XP_015067867.1 | 3263 | basic PR-P-like isoform X2 | 30.42 | 776.36 | 4.67 | 1.48E-44 |
| XP_008660804.1 | 1553 | basic PR-P-like | 0.44 | 34.62 | 6.29 | 1.30E-06 |
| XP_015067866.1 | 759 | basic PR-P-like isoform X1 | 0.97 | 21.88 | 4.49 | 4.62E-05 |
| XP_008660804.1 | 1574 | basic PR-P-like | 0.43 | 28.77 | 6.07 | 1.99E-06 |
| XP_015067867.1 | 1423 | basic PR-P-like isoform X2 | 10.74 | 285.83 | 4.73 | 7.99E-06 |
| XP_015067866.1 | 1560 | basic PR-P-like isoform X1 | 0.44 | 35.11 | 6.31 | 4.31E-07 |
| XP_008660804.1 | 860 | basic PR-P-like | 0.15 | 5.06 | 5.06 | 0.015 |
| XP_008660804.1 | 745 | basic PR-P-like | 16.93 | 5.44 | -1.64 | 0.052 |
| XP_015067866.1 | 4581 | basic PR-P-like isoform X1 | 97.94 | 596.69 | 2.61 | 7.26E-16 |
| XP_006362904.1 | 1353 | basic PR-P | 67.59 | 85.13 | 0.33 | 0.34 |
| XP_015067866.1 | 2379 | basic PR-P-like isoform X1 | 0.31 | 27.95 | 6.48 | 0.0011 |
| XP_015067866.1 | 441 | basic PR-P-like isoform X1 | 0.15 | 9.56 | 5.96 | 0.0018 |
| XP_008660804.1 | 1428 | basic PR-P-like | 15.82 | 284.06 | 4.17 | 1.40E-28 |
| XP_015067866.1 | 2282 | basic PR-P-like isoform X1 | 2.68 | 21.28 | 2.99 | 0.055 |
| XP_015067866.1 | 1063 | basic PR-P-like isoform X1 | 0.41 | 12.65 | 4.93 | 0.00060 |
| XP_015067866.1 | 2152 | basic PR-P-like isoform X1 | 2.27 | 43.92 | 4.27 | 1.38E-08 |
| XP_015067866.1 | 957 | basic PR-P-like isoform X1 | 0.19 | 45.82 | 7.94 | 1.60E-06 |
| XP_009103356.1 | 837 | anther-specific PR-P APG | 37.86 | 10.64 | -1.83 | 0.00080 |
| XP_014755416.1 | 1091 | basic PR-P-like | 0.13 | 0.24 | 0.87 | 0.70 |
| XP_015067866.1 | 1799 | basic PR-P-like isoform X1 | 8.62 | 47.23 | 2.45 | 9.99E-06 |
| XP_008660804.1 | 1136 | basic PR-P-like | 156.31 | 649.24 | 2.05 | 1.30E-21 |
| XP_014755416.1 | 1256 | basic PR-P-like | 423.93 | 205.23 | -1.05 | 8.06E-05 |
| XP_015067866.1 | 451 | basic PR-P-like isoform X1 | 0.44 | 17.14 | 5.27 | 0.00020 |
| XP_015067866.1 | 667 | basic PR-P-like isoform X1 | 1.21 | 38.41 | 4.98 | 1.67E-07 |
| XP_015067866.1 | 2218 | basic PR-P-like isoform X1 | 0.32 | 33.86 | 6.74 | 0.00051 |
| XP_015067867.1 | 1378 | basic PR-P-like isoform X2 | 0.97 | 33.95 | 5.12 | 8.80E-08 |
| XP_015067866.1 | 3037 | basic PR-P-like isoform X1 | 0.29 | 19.25 | 6.07 | 0.0027 |
| XP_015067866.1 | 2233 | basic PR-P-like isoform X1 | 1.57 | 179.66 | 6.84 | 4.24E-17 |
| XP_011657308.1 | 280 | basic salivary PR-P 2 | 0.55 | 1.97 | 1.83 | 0.47 |
| XP_015067866.1 | 2180 | basic PR-P-like isoform X1 | 0.99 | 62.24 | 5.98 | 3.47E-11 |
| XP_014755416.1 | 252 | basic PR-P-like | 0.15 | 3.94 | 4.74 | 0.026 |
| XP_006480264.1 | 625 | basic PR-P-like | 0.14 | 0.95 | 2.78 | 0.26 |
| XP_015067866.1 | 890 | basic PR-P-like isoform X1 | 0.21 | 70.63 | 8.40 | 2.67E-07 |
| XP_014755416.1 | 1293 | basic PR-P-like | 432.47 | 181.36 | -1.25 | 3.11E-05 |
| XP_008660804.1 | 1489 | basic PR-P-like | 2.26 | 45.88 | 4.35 | 4.73E-08 |
| XP_014755416.1 | 249 | basic PR-P-like | 0.13 | 0.24 | 0.87 | 0.70 |
| XP_008660804.1 | 728 | basic PR-P-like | 0.11 | 1.07 | 3.23 | 0.18 |
| XP_015067866.1 | 3713 | basic PR-P-like isoform X1 | 21.31 | 48.08 | 1.17 | 0.0053 |
| XP_015067866.1 | 711 | basic PR-P-like isoform X1 | 47.58 | 34.57 | -0.46 | 0.39 |
| XP_010477244.1 | 461 | anther-specific PR-P APG | 0.15 | 1.31 | 3.09 | 0.21 |
| XP_015067866.1 | 450 | basic PR-P-like isoform X1 | 36.26 | 0.61 | -5.89 | 4.23E-06 |
| XP_008663939.1 | 320 | basic PR-P-like | 0.13 | 0.23 | 0.85 | 0.70 |
| XP_014755416.1 | 736 | basic PR-P-like | 1.05 | 86.99 | 6.37 | 1.96E-09 |
| XP_015067866.1 | 1662 | basic PR-P-like isoform X1 | 6.71 | 111.37 | 4.05 | 1.94E-06 |
| XP_015067866.1 | 3513 | basic PR-P-like isoform X1 | 0.99 | 33.34 | 5.07 | 4.29E-06 |
| XP_008660804.1 | 1650 | basic PR-P-like | 2.24 | 21.98 | 3.29 | 0.00011 |
| XP_010477244.1 | 535 | anther-specific PR-P APG | 0.21 | 4.18 | 4.33 | 0.056 |
| XP_015067866.1 | 2147 | basic PR-P-like isoform X1 | 7.69 | 132.20 | 4.10 | 7.70E-05 |
| XP_015067866.1 | 4169 | basic PR-P-like isoform X1 | 11.21 | 274.30 | 4.61 | 3.95E-26 |
| XP_015067866.1 | 1562 | basic PR-P-like isoform X1 | 2.11 | 60.00 | 4.83 | 4.71E-10 |
| XP_008646858.1 | 867 | basic PR-P-like | 1.79 | 82.66 | 5.53 | 1.37E-15 |
| XP_015067866.1 | 1897 | basic PR-P-like isoform X1 | 66.07 | 699.21 | 3.40 | 4.80E-25 |
| XP_008660804.1 | 949 | basic PR-P-like | 2.02 | 45.59 | 4.50 | 3.63E-10 |
| XP_014755416.1 | 594 | basic PR-P-like | 0.15 | 1.31 | 3.09 | 0.21 |
| XP_014755416.1 | 1542 | basic PR-P-like | 132.09 | 69.78 | -0.92 | 0.0011 |
| XP_014755416.1 | 1441 | basic PR-P-like | 0.13 | 0.09 | -0.52 | 0.77 |
| XP_014755416.1 | 776 | basic PR-P-like | 68.55 | 27.05 | -1.34 | 0.0016 |
| XP_014755416.1 | 624 | basic PR-P-like | 0.16 | 9.84 | 5.99 | 0.0017 |
| XP_015067866.1 | 1614 | basic PR-P-like isoform X1 | 5726.81 | 982.72 | -2.54 | 1.76E-09 |
| XP_008660804.1 | 1932 | basic PR-P-like | 0.72 | 41.93 | 5.86 | 1.54E-07 |
| XP_008660804.1 | 1926 | basic PR-P-like | 19.75 | 95.24 | 2.27 | 0.20 |
| XP_015067866.1 | 1358 | basic PR-P-like isoform X1 | 135.05 | 203.49 | 0.59 | 0.15 |
| XP_015067866.1 | 2279 | basic PR-P-like isoform X1 | 1.84 | 80.20 | 5.44 | 6.34E-14 |
| XP_015067866.1 | 2241 | basic PR-P-like isoform X1 | 3.44 | 215.81 | 5.97 | 1.24E-23 |
| XP_014660311.1 | 394 | basic PR-P-like | 0.90 | 2.86 | 1.66 | 0.39 |
| XP_015067866.1 | 2081 | basic PR-P-like isoform X1 | 0.19 | 47.28 | 7.95 | 1.68E-06 |
| XP_015067866.1 | 3805 | basic PR-P-like isoform X1 | 20.95 | 1606.52 | 6.26 | 1.19E-15 |
| XP_010313594.1 | 310 | basic PR-P, partial | 9.09 | 1.70 | -2.42 | 0.043 |
| XP_008680864.1 | 637 | basic PR-P-like | 0.15 | 7.11 | 5.57 | 0.0047 |
| XP_008660804.1 | 1894 | basic PR-P-like | 16.47 | 527.81 | 5.00 | 1.09E-13 |
| XP_015067866.1 | 2107 | basic PR-P-like isoform X1 | 0.19 | 23.30 | 6.92 | 0.00013 |
| XP_015067866.1 | 666 | basic PR-P-like isoform X1 | 0.21 | 0.13 | -0.75 | 0.72 |
| XP_010313594.1 | 492 | basic PR-P, partial | 20.97 | 5.11 | -2.04 | 0.0034 |
| XP_006339345.1 | 982 | anther-specific PR-P APG-like | 1.44 | 12.87 | 3.16 | 0.063 |
| XP_015067866.1 | 3117 | basic PR-P-like isoform X1 | 20.85 | 1159.18 | 5.80 | 1.38E-09 |
| XP_006339345.1 | 2024 | anther-specific PR-P APG-like | 1141.39 | 23.88 | -5.58 | 1.03E-44 |
| XP_015067866.1 | 5570 | basic PR-P-like isoform X1 | 8.31 | 661.98 | 6.32 | 3.31E-35 |
| XP_014755416.1 | 225 | basic PR-P-like | 0.17 | 2.72 | 3.96 | 0.086 |
| XP_008660804.1 | 2060 | basic PR-P-like | 4.98 | 216.22 | 5.44 | 7.82E-28 |
| XP_010313594.1 | 247 | basic PR-P, partial | 7.28 | 2.05 | -1.83 | 0.10 |
| XP_015067866.1 | 3165 | basic PR-P-like isoform X1 | 18.44 | 1763.84 | 6.58 | 4.63E-44 |
| XP_010644788.1 | 876 | basic PR-P-like | 3.22 | 167.08 | 5.70 | 1.85E-14 |
| XP_008653760.1 | 399 | basic PR-P-like | 0.45 | 11.66 | 4.71 | 0.0020 |
